# Coffee Consumption is Associated with a High Prevalence of Headache in a Taiwanese Population Study

**DOI:** 10.7150/ijms.123794

**Published:** 2026-01-01

**Authors:** Pin-Rong Chen, Chih-Hsien Hung, Sun-Wung Hsieh, Chien-Hsun Li, Tzu-Chao Lin, Szu-Chia Chen, Kuo-Wei Lee

**Affiliations:** 1 Department of Medicine, Kaohsiung Medical University, Kaohsiung, Taiwan.; 2 Department of Neurology, Kaohsiung Medical University Hospital, Kaohsiung Medical University, Kaohsiung, Taiwan.; 3 School of Medicine, College of Medicine, Kaohsiung Medical University, Kaohsiung, Taiwan.; 4 Department of Public Health, Kaohsiung Medical University, Kaohsiung, Taiwan.; 5 Department of Neurology, Kaohsiung Municipal Siaogang Hospital, Kaohsiung Medical University, Kaohsiung, Taiwan.; 6 Department of Neurology, Faculty of Medicine, College of Medicine, Kaohsiung Medical University, Kaohsiung, Taiwan.; 7 Neuroscience Research Center, Kaohsiung Medical University, Kaohsiung, Taiwan.; 8 Department of Internal Medicine, Kaohsiung Municipal Siaogang Hospital, Kaohsiung Medical University Hospital, Kaohsiung Medical University, Kaohsiung, Taiwan.; 9 Division of Nephrology, Department of Internal Medicine, Kaohsiung Medical University Hospital, Kaohsiung Medical University, Taiwan.; 10 Faculty of Medicine, College of Medicine, Kaohsiung Medical University, Kaohsiung, Taiwan.

**Keywords:** coffee consumption, dietary factors, headache, Taiwan Biobank

## Abstract

The relationship between coffee consumption and headache remains controversial. While caffeine has both analgesic and vasoconstrictive properties, excessive intake is associated with a higher prevalence of headache. This cross-sectional study investigated the association between coffee consumption and headache in a large Taiwanese cohort. Data were obtained from 27,109 participants aged 30-70 years from the Taiwan Biobank. Headache status and coffee consumption patterns, including type, frequency, and daily intake, were assessed via structured questionnaires. Multivariable logistic regression models were used to evaluate associations. Among participants, 19.7% reported headaches. Coffee consumption was significantly associated with increased odds of headache (odds ratio = 1.21; 95% confidence level= 1.14-1.29; p < 0.001). All coffee types, including black coffee, coffee with non-dairy creamer, and coffee with milk, were linked to elevated headache risk. Daily intake of one, two, or ≥ three cups was also associated with higher odds. Frequent coffee consumption (daily or weekly) is linked to higher odds of headache, whereas monthly consumption is not. Subgroup analyses revealed no significant associations between coffee consumption and headache in individuals aged ≥ 65 years or with diabetes, hypertension, depression, or a history of alcohol or tea consumption. These findings suggest that both the amount and frequency of coffee intake are associated with higher occurrence of headache, emphasizing the importance of personalized caffeine recommendations, particularly for individuals prone to headaches.

## Introduction

Headache is one of the most prevalent neurological disorders affecting 52% of the global population annually, and subtypes include migraine (14%), tension-type headache (26%), and chronic headache (4.6%) 1. These primary headache disorders impose a significant global health burden 2. The Global Burden of Disease 2015 reported that headache disorders contribute more to disability-adjusted life years than all other neurological disorders combined, and that it ranks sixth among causes of years lived with disability 3. Risk factors for primary headaches encompass both genetic and environmental factors 4. Common environmental triggers include stress and sleep disturbances, particularly in tension-type headaches and migraines 5. Hormonal fluctuations, especially in estrogen levels, are known to be associated with migraines in women 6, and lifestyle factors such as irregular eating patterns, dehydration, and excessive caffeine consumption or withdrawal have also been associated with the risk of headache 7.

Coffee is a major dietary source of caffeine and it has been studied extensively for its diverse health effects. High coffee intake (≥ 4 cups daily) has been associated with lower risks of cardiometabolic conditions including type 2 diabetes mellitus (DM) 8, ischemic heart disease 9, and hypertension 10, as well as gastrointestinal and metabolic conditions such as ulcerative colitis 11, liver cirrhosis 12 and gout 13. A recent study reported that individuals with a morning-type (4 a.m. to 11:59 a.m.) coffee consumption pattern were associated with lower risks of all-cause and cardiovascular mortality compared to non-coffee drinkers 14. However, excessive coffee intake has been linked to adverse effects including anxiety 15, insomnia 16 and gastrointestinal disorders 17.

The relationship between coffee consumption and headache remains controversial 18, 19. Some studies have reported that caffeine may relieve headaches by antagonizing adenosine receptors, thereby preventing the inhibitory effects of adenosine on neuronal activity 18. Caffeine has been shown to exert an analgesic effect by independently blocking adenosine A2A receptors, consequently relieving certain headaches 18. In a Korean nationwide cross-sectional survey, migraine sufferers with higher coffee consumption reported lower levels of depression and stress, with no significant differences in clinical headache characteristics or treatment response 20. Conversely, other studies have not shown a causal relationship between coffee consumption and migraine risk 21. Some evidence has even suggested that excessive or habitual caffeine use may increase headache frequency 22. A study using data from the National Health and Nutrition Examination Survey (NHANES) found that higher caffeine intake was associated with more severe headaches or migraines in United States adults. In addition, for every 100 mg increase in daily caffeine intake the risk increased by 5%, and people who consumed 400 mg or more per day had a 42% higher risk compared to those who consumed 0-40 mg per day 22. Furthermore, caffeine withdrawal has been shown to further complicate the relationship between coffee and headache, and sudden cessation after prolonged intake may result in rebound cerebral vasodilation, triggering withdrawal headaches due to upregulation of adenosine receptors 23.

This study aimed to elucidate the relationship between coffee consumption and headache using a large dataset of over 27,000 participants from the Taiwan Biobank (TWB). We hypothesized that coffee consumption, including the type, frequency, and daily intake, may influence the prevalence of headache. Furthermore, we explored these associations across various demographic subpopulations to provide nuanced insights into the role of coffee in the pathophysiology of headache and its potential implications for prevention and management strategies.

## Materials and Methods

### Study population and data source

We used data from the TWB, a nationwide cohort initiated in 2008 to collect health-related information from Taiwanese adults. The TWB contains demographic, lifestyle, clinical and genetic data 24, 25. The project received ethical approval from the Institutional Review Board on Biomedical Science Research at Academia Sinica and is overseen by the TWB Ethics and Governance Council.

**Figure 1:**
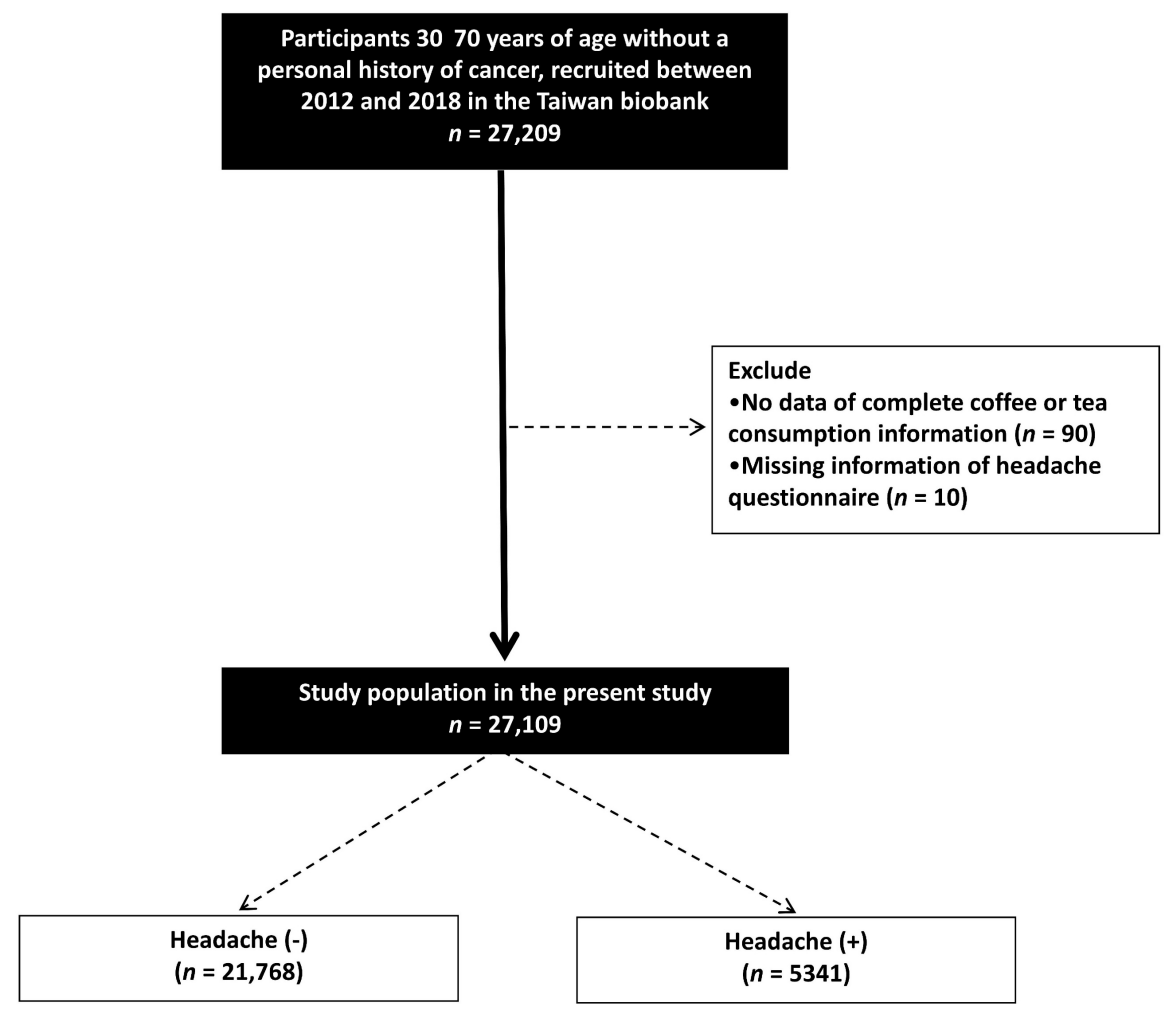
Flowchart of study population.

This study recruited 27,209 participants aged 30 to 70 years without a personal history of cancer from the TWB between 2012 and 2018. Participants with incomplete information regarding coffee or tea consumption (*n* = 90) and those who did not complete a headache questionnaire (*n* = 10) were excluded. Consequently, a total of 27,109 participants were included in the final analysis (Figure 1).

### Ethics statement

All enrollees in the TWB are requested to sign written informed consent forms. The current study was approved by the Institutional Review Board of Kaohsiung Medical University Hospital (KMUHIRB-E(I)-20210058) and was conducted following the Helsinki Declaration.

### Collection of study variables

Demographic data including age, sex, and educational attainment were collected, and data on lifestyle factors such as smoking history, alcohol consumption, and coffee and tea consumption were obtained through structured questionnaires. Regular exercise was defined as performing any kind of physical activity for ≥ 30 minutes ≥ 3 times a week 26. Histories of hypertension, DM and depression were also collected. We also recorded height, weight, and body mass index as kg/m^2^. Blood pressure (BP) was measured three times after a 1-2-minute rest using a standardized electronic BP device, following at least 30 minutes of refraining from smoking, caffeine intake or exercise. Other variables of interest included fasting glucose, hemoglobin, triglycerides, total cholesterol, high-density lipoprotein cholesterol (HDL-C), low-density lipoprotein cholesterol (LDL-C), uric acid, and estimated glomerular filtration rate (eGFR, calculated using a previously reported method 27).

### Headache assessment

The participants were asked to complete a questionnaire assessing the presence, frequency, and severity of headache or migraine symptoms. Those who reported experiencing headaches or migraines were classified into the with headache group, and the others were classified into the without headache group.

### Coffee consumption assessment

The participants were also asked whether they consumed coffee regularly; those who stated that they did were classified as coffee drinkers, and the others were classified as non-coffee drinkers. The coffee drinkers were then asked the following questions:

1. “What type of coffee do you usually drink?” The participants were then classified into the following groups accordingly: “black coffee”, “coffee with creamer” or “coffee with milk".

2. “How many cups of coffee (one cup = 237 mL) do you typically consume daily?” The participants were then classified into “none”, “one cup per day”, “two cups per day” or “three cups or more per day" groups.

3. “How often do you drink coffee?” The participants were then classified into “never”, “every day”, “weekly” (frequency less than every day), and “monthly” (frequency less than weekly) groups.

### Statistical analysis

Descriptive statistics were presented as means ± standard deviation or percentages. Group differences were analyzed using the independent t-test (continuous variables) and chi-square test (categorical variables). Multivariable logistic regression was conducted to identify associations between coffee consumption (type, intake and frequency) with headache, adjusting for covariates. Significant variables in univariable analysis were used for multivariable analysis. A two-tailed *p*-value < 0.05 was considered statistically significant. Analyses were performed using SPSS version 25 (IBM Inc., Armonk, NY).

## Results

Of the 27,109 participants, 21,768 (80.3%) were classified into the without headache group and 5341 (19.7%) into the with headache group.

### Comparison of clinical characteristics between the headache groups

The clinical characteristics of the participants with and without headache are presented in Table 1. The participants with headache were younger (mean age: 51.0 ± 10.1 years *vs.* 56.0 ± 10.1 years; *p* < 0.001), more likely to be female (77.8% *vs.* 61.4%; *p* < 0.001), and have a higher rate of depression (6.3% *vs.* 3.6%; *p* < 0.001) than those without headache. They also had lower rates of DM (5.7% *vs.* 8.2%; *p* < 0.001), hypertension (13.1% *vs.* 19.1%; *p* < 0.001), smoking history (21.2% *vs.* 26.7%; *p* < 0.001), alcohol history (6.8% *vs.* 11.6%; *p* < 0.001), and tea consumption (22.2% *vs.* 24.1%; *p* = 0.003), while having a higher rate of coffee consumption (45.9% *vs.* 40.0%; *p* < 0.001). In addition, the participants with headache had a lower prevalence of regular exercise habits (36.9% *vs.* 51.1%; *p* < 0.001) and a higher level of education (*p* < 0.001).

Regarding physiological parameters, the participants with headache had significantly lower systolic and diastolic BP (*p* < 0.001), body mass index (*p* < 0.001), fasting glucose (*p* < 0.001), hemoglobin (*p* < 0.001), triglycerides (*p* = 0.004) and uric acid (*p* < 0.001), but higher total cholesterol (*p* = 0.001), HDL-C (*p* < 0.001), LDL-C (*p* = 0.005) and eGFR (*p* < 0.001) compared to those without headache.

### Associations between coffee consumption and other factors with headache

The results of multivariable logistic regression analysis for the association between coffee consumption and other factors with headache are shown in Table 2. Coffee consumption was significantly associated with an increased odds of headache (odds ratio [OR] = 1.209; 95% confidence interval [CI] = 1.135-1.288; *p* < 0.001). Depression (OR = 1.814; 95% CI = 1.581-2.082; *p* < 0.001) and a history of smoking (OR = 1.189; 95% CI = 1.085-1.305; *p* < 0.001) were also significantly associated with higher odds of headache. In contrast, older age (OR = 0.952; 95% CI = 0.948-0.957; *p* < 0.001), male sex (*vs.* female; OR = 0.437; 95% CI = 0.392-0.487; *p* < 0.001), alcohol history (OR = 0.810; 95% CI = 0.751-0.872; *p* < 0.001), and regular exercise (OR = 0.764; 95% CI = 0.714-0.817; *p* < 0.001) were associated with lower odds of headache. Educational attainment also showed an inverse pattern, with individuals who completed middle to high school education (OR = 0.853; 95% CI = 0.744-0.978; *p* = 0.027) and those with a college degree or higher (OR = 0.777; 95% CI = 0.674-0.896; *p* = 0.001) had a significantly lower odds of headache compared to those with an elementary-level education or less.

In terms of physiological parameters, systolic BP (per 1 mmHg) was inversely associated with headache odds (OR = 0.994; 95% CI = 0.991-0.997; *p* < 0.001), while diastolic BP (per 1 mmHg) showed a slight positive association (OR = 1.014; 95% CI = 1.010-1.019; *p* < 0.001). Body mass index (per 1 kg/m²) was modestly associated with a lower odd of headache, with an OR of 0.979 (95% CI = 0.969-0.989; *p* < 0.001).

Among the laboratory markers, HDL-C (per 1 mg/dL) was associated with lower odds of headache (OR = 0.994; 95% CI = 0.990-0.998; *p* = 0.006). Likewise, higher eGFR (OR = 0.997, 95% CI = 0.994-0.999, *p* = 0.013) and uric acid (OR = 0.961, 95% CI = 0.933-0.991, *p* = 0.007) were both modestly inversely associated with the odds of headache.

### Association between coffee consumption patterns (type, daily intake and frequency) with headache

The results of analysis of the association between coffee consumption patterns (type, daily intake and frequency) with headache are shown in Table 3. Compared to non-coffee drinkers, all types of coffee were significantly associated with an increased odds of headache: black coffee (OR = 1.166; 95% CI = 1.080-1.258), coffee with non-dairy creamer (OR = 1.182; 95% CI = 1.039-1.344), and coffee with milk (OR = 1.311; 95% CI = 1.190-1.444) (all *p* < 0.05). Daily consumption was classified into three categories based on a standard cup size of 237 mL: one cup per day (237 mL), two cups per day (474 mL), and three cups per day (711 mL) per day. Notably, those who drank one, two and three cups per day were all associated with an increased odds of headache, with those who drank two cups per day having the highest odds (OR = 1.254; 95% CI = 1.166-1.349; *p* < 0.001). In addition, analysis of frequency indicated that consuming coffee daily (OR = 1.212; 95% CI = 1.125-1.306; *p* < 0.001) or weekly (OR = 1.206; 95% CI = 1.108-1.312; *p* < 0.001) was significantly associated with greater odds of headache, while monthly consumption was not (*p* = 0.314).

### Subgroup analyses: association between coffee consumption and other factors with headache

The association between coffee consumption and headache across various demographic and clinical factors are shown in Table 4. No significant associations were observed in those with older age (≥ 65 years old), DM, hypertension, depression, or a history of alcohol or tea consumption.

## Discussion

In this community-based Taiwanese cross-sectional study, we found that habitual coffee consumption was significantly associated with a higher prevalence of headaches, whereas tea consumption showed no significant correlation. Both the amount and frequency of coffee consumption showed associations with headache prevalence. In addition, subgroup analyses showed no significant associations in older adults (≥ 65 years), individuals with DM, hypertension, or depression, or those with a history of alcohol or tea consumption. These findings highlight the importance of considering individual patterns of coffee consumption when examining headache prevalence.

The key finding of this study is that coffee consumption was associated with higher odds of headache. Previous cellular, animal and clinical studies have provided insights into the potential mechanisms underlying this relationship. At the cellular level, elevated adenosine levels have been linked to migraine attacks, with exogenous adenosine potentially triggering such attacks and inhibition of adenosine uptake increasing their frequency 28, 29. Adenosine activates receptors including A1 and A3 (vasoconstriction) and A2A and A2B (vasodilation), which has been shown to influence blood vessel function during migraine attacks 28. Caffeine, a non-selective adenosine receptor antagonist, has been shown to block multiple adenosine receptors, including A1, A2A, and A2B, thereby disrupting the vascular changes linked to migraines 28. Caffeine-induced adenosine receptor antagonism can cause transient vasoconstriction followed by rebound vasodilation, which may contribute to migraine onset 18. Furthermore, the chronic activation of A2A receptors by excessive caffeine intake has been shown to promote the action of calcitonin gene-related peptide (CGRP), leading to increased pain signaling and contributing to the development of chronic headaches 23. Animal studies further support these findings 28. Activation of A2A receptors has been demonstrated to enhance the action of CGRP and increase pain signaling in rat hippocampi and also in the rat trigeminovascular system, where A2A receptors are expressed 28. In addition, chronic and excessive caffeine intake can lead to the upregulation of A2A receptors, amplifying the pain signaling pathway mediated by CGRP 23, 28. Furthermore, a study on Korean nurses also identified coffee consumption as the most significant migraine trigger 30.

In contrast to coffee consumption, our results showed that tea consumption was not associated with headache. This may be partly explained by the presence of polyphenols, which are bioactive compounds abundant in green tea. These compounds are thought to reduce oxidative stress and neuroinflammation, both of which are implicated in migraine pathophysiology 31. A previous study suggested that diets rich in polyphenols may lower migraine severity and disability, with specific polyphenols such as flavanones and lignans showing inverse correlations with migraine intensity 31. Supporting these observations, a Mendelian randomization study using data from the United Kingdom Biobank and the International Headache Genetics Consortium also found no causal relationship between tea intake and migraine, including subtypes such as migraine with and without aura 32.

Another important finding of this study is that both the amount and frequency of coffee consumption were associated with higher odds of headache. Our findings suggest a potential dose-related association, with the highest odds observed among individuals consuming two cups daily. However, although daily and weekly consumption were associated with a significantly increased risk of headache, monthly intake was not. Notably, the 'per month' frequency category contained only 15 participants, resulting in an extremely wide confidence interval (OR = 0.345; 95% CI = 0.044-2.734). This finding should therefore be interpreted with considerable caution. The lack of statistical significance in this subgroup does not rule out a meaningful association and is likely attributable to limited statistical power due to the very small sample size. Previous studies support these findings. A recent cohort study using data from the Korea Nurses' Health Study analyzed the risk of migraine based on lifestyle and psychological factors, and found that drinking three or more cups/day increased the risk by 1.67 times (95% CI = 1.18-2.36), while drinking fewer than three cups/day increased the risk by 1.44 times (95% CI = 1.05-1.97) compared to non-coffee drinkers 30. These results suggest that both low and high coffee consumption is correlated with a higher risk of migraine, and that higher intake may be linked to an even greater risk 30. Another study reported a nonlinear relationship between caffeine intake and migraine risk, where low to moderate doses (1-2 servings of caffeinated beverages) did not increase the risk, but high doses (3 or more servings) significantly increased the risk 33. In addition, the study also found an association between the frequency and amount of caffeine intake with the risk of migraine 33. Individuals with lower habitual intake experienced migraines at lower doses, whereas those with higher regular intake had an increased risk only at three or more servings per day, suggesting that regular caffeine intake may build tolerance to its effects 33. These findings align with our study in that the impact of coffee consumption on headaches was influenced by both dose and frequency.

Our subgroup analyses revealed that in certain populations, coffee consumption was not associated with higher headache prevalence. We found no significant association in older adults, which could reflect age-related changes in caffeine metabolism and reduced headache sensitivity. Previous research has shown that caffeine absorption remains unchanged in older individuals, but that its volume of distribution is reduced 34. This may result in a more localized effect and lower systemic impact of caffeine in older adults. Furthermore, the elimination rate of caffeine has been shown to be slightly faster in older adults, indicating a potential adaptation or increased clearance efficiency with age 34. There is also evidence indicating reduced headache sensitivity with age, and several clinical studies have observed that older age is not associated with an increased prevalence of developing headaches or migraines 35, 36. A study conducted in Singapore also found that individuals aged 65 years and older were less likely to have migraines compared to those aged 18-34, with the prevalence ratio for the older age group being 0.44 (95% CI = 0.21-0.93) 35.

Similarly, we found no significant association between coffee consumption and headache prevalence among individuals with DM or hypertension. Although the underlying mechanism remains unclear, coffee contains bioactive compounds with anti-inflammatory and antioxidative properties. These compounds have been hypothesized to counteract chronic low-grade inflammation and vascular dysfunction, which are commonly observed in individuals with DM and hypertension, and they may also play a role in the pathophysiology of headache 37. A meta-analysis of 30 prospective studies encompassing 1,185,210 participants and 53,018 incident cases demonstrated an inverse association between coffee consumption and the risk of type 2 diabetes (T2D). A dose-response relationship was observed, indicating a 6% reduction in T2D risk per additional cup of coffee consumed daily (relative risk [RR] = 0.94; 95% CI = 0.93-0.95) 37. Another longitudinal analysis from the Nord-Trondelag Health Survey examined the relationship between BP and headache development 38. The study identified an inverse association between baseline BP and headache, with higher systolic BP linked to a reduced risk of developing any headache subtype 38. These findings suggest an inverse relationship between BP and the odds of headache.

We also found that individuals with depression had a higher prevalence of headache, which is consistent with the known comorbidity between migraines and mood disorders. However, coffee consumption was not associated with higher odds of headache in this population. One possible explanation is that the mood of individuals with depression and headaches may be improved by coffee consumption, which may in turn modulate headache perception or frequency. A previous meta-analysis demonstrated a significant inverse association between coffee consumption and the risk of depression 39. A Korean study also reported that migraine sufferers who drank more coffee had lower levels of depression and stress 20.

We also found that the individuals who habitually consumed tea did not show evidence of a higher likelihood of headache in relation to coffee consumption. This may indicate an association between prior caffeine exposure and susceptibility to headache, and may also suggest that regular tea drinkers develop caffeine tolerance, which could reduce the likelihood of caffeine-related headaches. Supporting this hypothesis, a prospective cohort study found that individuals with low habitual caffeine intake were more likely to have migraines even after consuming only 1-2 servings of caffeinated beverages, whereas those who regularly consumed caffeinated beverages had an increased headache risk only when consuming ≥ 3 servings per day 33.

Our results also showed a negative association between alcohol consumption and the prevalence of headache, even after accounting for coffee consumption. This inverse association is supported by recent population-based studies. A large-scale analysis of NHANES data from 13,083 adults revealed that increasing dietary alcohol intake was associated with lower odds of migraine or severe headache 40. Similarly, a recent meta-analysis including over 126,000 participants reported that alcohol drinkers had a significantly lower risk of migraine compared to non-drinkers (RR = 0.71; 95% CI = 0.57-0.89) 41.​ However, these findings should be interpreted with caution, as it may reflect reverse causality, which cannot be ruled out by our cross-sectional design. Individuals who suffer from recurrent headaches may deliberately limit or avoid alcohol consumption to prevent symptom exacerbation, which could result in an apparent protective association in cross-sectional analyses.

There are important strengths to our study, including the large community-based sample of healthy individuals with no history of cancer, and the detailed data on coffee consumption patterns. We adjusted for numerous confounders and examined subgroup effects. However, there are also several limitations. First, the diagnosis of headache was based on self-reported data from questionnaires without clinical verification or classification into specific headache disorders. As a result, our headache outcome represents a heterogeneous group of disorders rather than specific subtypes (e.g., migraine, tension-type headache, cluster headache), which is a major limitation and may dilute or obscure subtype-specific associations with coffee consumption. Nevertheless, a previous study demonstrated moderate concordance between claims records and self-reported health conditions in Taiwan, suggesting that self-reported data can be a reliable source in certain contexts 42. Second, this was a cross-sectional study, and we did not evaluate for how long the participants had been having headaches. Therefore, we could not evaluate the causal relationship between coffee consumption and headache. Further longitudinal studies are warranted to investigate the risk of incident headache. Third, we were unable to determine whether a specific threshold of coffee consumption exists beyond which its association with headache differs, because some individuals may avoid higher intake due to side effects related to caffeine and tannin contents. Fourth, the collection of information on habitual coffee consumption relied on self-reported questionnaires, which may have introduced recall bias. Finally, the participants in this study were of Chinese ethnicity, and thus our findings may not be generalizable to other ethnicities.

## Conclusion

This large-scale population study demonstrated that coffee consumption was associated with a higher prevalence of headache in Taiwanese adults. The association varied by both the amount and frequency of consumption. Notably, this association was not observed in older adults or individuals with chronic conditions such as DM, hypertension, or depression, indicating potential variations in individual susceptibility. These findings suggest that habitual coffee intake may be associated with headache prevalence. Prospective studies are needed to clarify whether modifying individual patterns of caffeine consumption has clinical relevance for headache prevention and management.

## Figures and Tables

**Table 1:** Comparison of clinical characteristics among participants according to headache in study participants

Characteristics	Headache (-) (*n* = 21,768)	Headache (+) (*n* = 5341)	*p*
Age (year)	56.0 ± 10.1	51.0 ± 10.1	< 0.001
Male sex (%)	38.6	22.2	< 0.001
DM (%)	8.2	5.7	< 0.001
Hypertension (%)	19.1	13.1	< 0.001
Depression (%)	3.6	6.3	< 0.001
Smoking history (%)	26.7	21.2	< 0.001
Alcohol history (%)	11.6	6.8	< 0.001
Tea consumption (%)	24.1	22.2	0.003
Coffee consumption (%)	40.0	45.9	< 0.001
Regular exercise habits (%)	51.1	36.9	< 0.001
Education status			
≤ Elementary (%)	7.7	5.8	< 0.001
Middle to high school (%)	44.1	43.0	
≥ College (%)	48.2	51.2	
Systolic BP (mmHg)	125.8 ± 19.5	120.9 ± 18.9	< 0.001
Diastolic BP (mmHg)	74.5 ± 11.3	73.3 ± 11.4	< 0.001
Body mass index (kg/m^2^)	24.4 ± 3.6	24.0 ± 3.8	< 0.001
Laboratory parameters			
Fasting glucose (mg/dL)	97.9 ± 22.1	95.4 ± 20.3	< 0.001
Hemoglobin (g/dL)	13.8 ± 1.5	13.4 ± 1.6	< 0.001
Triglyceride (mg/dL)	120.9 ± 99.9	116.7 ± 83.7	0.004
Total cholesterol (mg/dL)	195.8 ± 36.4	197.6 ± 35.4	0.001
HDL-C (mg/dL)	54.2 ± 13.5	55.5 ± 13.5	< 0.001
LDL-C (mg/dL)	119.9 ± 32.0	121.3 ± 31.4	0.005
eGFR (mL/min/1.73 m^2^)	96.9 ± 15.1	102.3 ± 14.5	< 0.001
Uric acid (mg/dL)	5.5 ± 1.4	5.2 ± 1.3	< 0.001

Abbreviations. DM, diabetes mellitus; BP, blood pressure; HDL-C, high-density lipoprotein cholesterol; LDL-C, low-density lipoprotein cholesterol; eGFR, estimated glomerular filtration rate.

**Table 2:** The association between coffee consumption and other factors with headache using multivariable logistic regression analysis

Variables	Multivariable (headache)	
	OR (95% CI)	*p*
Age (per 1 year)	0.952 (0.948-0.957)	< 0.001
Male sex (*vs.* female)	0.437 (0.392-0.487)	< 0.001
DM	1.079 (0.926-1.256)	0.245
Hypertension	1.050 (0.950-1.160)	0.286
Depression	1.814 (1.581-2.082)	< 0.001
Smoking history	1.189 (1.085-1.305)	< 0.001
Alcohol history	0.810 (0.751-0.872)	< 0.001
Tea consumption	0.989 (0.917-1.067)	0.599
Coffee consumption	1.209 (1.135-1.288)	< 0.001
Regular exercise habits	0.764 (0.714-0.817)	< 0.001
Education status		
≤ Elementary	Reference	
Middle to high school	0.853 (0.744-0.978)	0.027
≥ College	0.777 (0.674-0.896)	0.001
Systolic BP (per 1 mmHg)	0.994 (0.991-0.997)	< 0.001
Diastolic BP (per 1 mmHg)	1.014 (1.010-1.019)	< 0.001
Body mass index (per 1 kg/m^2^)	0.979 (0.969-0.989)	< 0.001
Laboratory parameters		
Fasting glucose (per 1 mg/dL)	1.000 (0.998-1.001)	0.698
Hemoglobin (per 1 g/dL)	1.007 (0.982-1.033)	0.511
Triglyceride (per 1 mg/dL)	1.000 (0.999-1.000)	0.789
Total cholesterol (per 1 mg/dL)	1.003 (1.000-1.006)	0.071
HDL-C (per 1 mg/dL)	0.994 (0.990-0.998)	0.006
LDL-C (per 1 mg/dL)	0.999 (0.995-1.002)	0.507
eGFR (per 1 mL/min/1.73 m^2^)	0.997 (0.994-0.999)	0.013
Uric acid (per 1 mg/dL)	0.961 (0.933-0.991)	0.007

Values expressed as odds ratio (OR) and 95% confidence interval (CI). Abbreviations are the same as in Table 1.

**Table 3:** Association of content, daily cups and frequency of coffee consumption with headache using multivariable regression analysis

Coffee consumption	Multivariable (headache)	
	OR (95% CI)	*p*
Coffee content		
Non-coffee (*n* = 15,955)	Reference	
Black coffee (*n* = 6605)	1.166 (1.080-1.258)	< 0.001
Coffee with non-dairy creamer (*n* = 1666)	1.182 (1.039-1.344)	0.011
Coffee with milk (*n* = 2893)	1.311 (1.190-1.444)	< 0.001
Daily cups		
None (*n* = 15,955)	Reference	
1 cup* per day (*n* = 3204)	1.127 (1.018-1.248)	0.022
2 cups* per day (*n* = 6779)	1.254 (1.166-1.349)	< 0.001
≥ 3 cups* per day (*n* = 1181)	1.166 (1.008-1.349)	0.039
Frequency		
None (*n* = 15,955)	Reference	
Per day (*n* = 6618)	1.212 (1.125-1.306)	< 0.001
Per week (*n* = 4531)	1.206 (1.108-1.312)	< 0.001
Per month (*n* = 15)	0.345 (0.044-2.734)	0.314

Values expressed as odds ratio (OR) and 95% confidence interval (CI). Abbreviations are the same as in Table 1. Adjusted for age, sex, DM, hypertension, depression, smoking and alcohol history, tea consumption, regular exercise habits, education status, systolic and diastolic BPs, body mass index, hemoglobin, triglyceride, total cholesterol, HDL-C, LDL-C, eGFR and uric acid. * One cup = 0.237 liter

**Table 4:** Subgroup analysis: the association between coffee consumption and headache

Subgroup	Headache OR (95% CI)	*p*
Age		
< 65 years (*n* = 21,555)	1.219 (1.139-1.304)	< 0.001
≥ 65 years (*n* = 5554)	1.137 (0.942-1.372)	0.182
Sex		
Male (*n* = 9588)	1.240 (1.092-1.408)	0.001
Female (*n* = 17,521)	1.199 (1.114-1.290)	< 0.001
DM		
Yes (*n* = 2089)	0.987 (0.758-1.284)	0.921
No (*n* = 25,020)	1.226 (1.149-1.309)	< 0.001
Hypertension		
Yes (*n* = 4848)	0.974 (0.820-1.158)	0.768
No (*n* = 22,261)	1.247 (1.165-1.335)	< 0.001
Depression		
Yes (*n* = 1122)	0.938 (0.713-1.234)	0.648
No (*n* = 25,997)	1.227 (1.150-1.310)	< 0.001
Smoking		
Yes (*n* = 6942)	1.197 (1.044-1.372)	0.010
No (*n* = 20,167)	1.209 (1.126-1.299)	< 0.001
Alcohol		
Yes (*n* = 2885)	1.162 (0.801-1.658)	0.429
No (*n* = 24,224)	1.199 (1.123-1.281)	< 0.001
Tea consumption		
Yes (*n* = 6427)	0.992 (0.868-1.134)	0.906
No (*n* = 20,682)	1.286 (1.197-1.382)	< 0.001
Regular exercise habits		
Yes (*n* = 13,088)	1.269 (1.148-1.404)	< 0.001
No (*n* = 14,021)	1.174 (1.081-1.273)	< 0.001

Values expressed as odds ratio (OR) and 95% confidence interval (CI). Abbreviations are the same as in Table 1. Adjusted for age, sex, DM, hypertension, depression, smoking and alcohol history, tea consumption, regular exercise habits, education status, systolic and diastolic BPs, body mass index, hemoglobin, triglyceride, total cholesterol, HDL-C, LDL-C, eGFR and uric acid.

## Abbreviations

BP: blood pressure
CI: confidence interval
CGRP: calcitonin gene-related peptide
DM: diabetes mellitus
eGFR: estimated glomerular filtration rate
HDL-C: high-density lipoprotein cholesterol
LDL-C: low-density lipoprotein cholesterol
NHANES: National Health and Nutrition Examination Survey
OR: odds ratio
RR: relative risk
T2D: type 2 diabetes
TWB: Taiwan Biobank
